# Pycnogenol^®^ and *Ginkgo biloba* extract: effect on peroxynitrite-oxidized sarcoplasmic reticulum Ca^2+^-ATPase

**DOI:** 10.2478/v10102-010-0053-8

**Published:** 2010-12

**Authors:** Petronela Žižková, Jana Viskupičová, L'ubica Horáková

**Affiliations:** Institute of Experimental Pharmacology & Toxicology, Slovak Academy of Sciences, SK-84104 Bratislava, Slovakia

**Keywords:** Ca^2+^-ATPase, EGb 761, Pycnogenol^®^, peroxynitrite

## Abstract

The effect of two natural standardized plant extracts, Pycnogenol^®^ and EGb 761, on sarcoplasmic reticulum Ca^2+^-ATPase (SERCA) activity and posttranslational modifications induced by peroxynitrite was investigated to assess their possible protective role. EGb 761 was found to have a protective effect on SERCA activity in the concentration range of 5–40 µg/ml. On the other hand, Pycnogenol^®^ caused a decrease of SERCA activity at concentrations of 25 µg/ml. EGb 761 did not prevent protein carbonyl formation upon oxidation with peroxynitrite. On the contrary, Pycnogenol^®^ at the concentrations of 5 and 10 µg/ml significantly decreased the level of protein carbonyls by 44% and 54%, respectively. Neither Pycnogenol^®^ nor EGb 761 exerted a protective effect against thiol group oxidation.The plant extracts studied modulated peroxynitrite-injured SERCA activity by different ways and failed to correlate with posttranslational modifications. Their effect seems to be associated with their ability to change SERCA conformation rather than by their antioxidant capacity.

**LIST OF ABBREVIATIONS**
3-NT: 3-nitrotyrosine
BHT: t-butylhydroxytoluene
**Ca**^**2 + **^: calcium ions
Cys: cysteine
DNPH: 2,4-dinitrophenylhydrazine
EGb 761: extract from leaves of *Ginkgo biloba*
ELISA: enzyme-linked immunosorbent assay
**IC**_**50**_: half maximal inhibitory concentration
·NO: nitric oxide
**O**^**2**^·–
				: superoxide anion radical
**ONOO**^–^: peroxynitrite
ROS: reactive oxygen species
SERCA: sarcoplasmic/endoplasmic reticulum Ca^2+^-ATPase
SR/ER: sarcoplasmic/endoplasmic reticulum
TEMPOL: 4-hydroxy-2,2,6,6-tetramethylpiperidine-1-oxyl

## Introduction

Recent evidence indicates that most of the cytotoxicity attributed to excessive nitric oxide (·NO) production is due to the activities of the peroxynitrite anion (ONOO^–^), which is a product of the diffusion-controlled reaction of ·NO with the superoxide anion radical (O_2_·^−^) (Szabo, 2007; Pacher, 2007). Several potentially harmful modifications are produced by peroxynitrite-derived radicals, including nitration of accessible tyrosine residues to form 3-nitrotyrosine (3-NT) or nitration of tryptophan residues, S-nitrosylation of cysteine (Cys) residues to form S-nitrosocysteine, oxidation of methionine (Groves, 1999), formation of protein carbonyls, and peroxidation of unsaturated fatty acid containing phospholipids (Eiserich *et al*., 1998; Szabo, 2003).

The sarcoplasmic/endoplasmic reticulum Ca^2+^-ATPase (SERCA) plays an important role in maintaining intracellular Ca^2+^ homeostasis through its ability to pump cytosolic Ca^2+^ into the sarcoplasmic/endoplasmic reticulum (SR/ER) (Tong *et al*., 2008). Under physiological conditions, nitric oxide stimulates SERCA activity and uptake of cytosolic Ca^2+^ via SERCA to relax vascular smooth muscle by direct modification of SERCA protein (Tong *et al*., 2010). Nitric oxide function is impaired in a variety of cardiovascular diseases, diabetes, hypercholesterolemia, and atherosclerosis, which are all associated with SERCA dysfunction caused by the increased levels of oxidants in these diseases (Pacher *et al*., 2007). Exposure of cells to large amounts of peroxynitrite *in vitro* leads rapidly to necrosis, due to the disruption of cellular metabolism and membrane integrity. The same may occur *in vivo* when large amounts of peroxynitrite are formed, as *e.g.* during ischemia-reperfusion or inflammation (Murphy *et al*., 1999). Peroxynitrite has been shown to inhibit avariety of ion pumps including calcium pumps, calcium-activated potassium channels and also membrane Na/K-ATPase activity (Szabó, 2003).

SERCA may serve as a therapeutic target to prevent diseases associated with oxidative stress. Synthetic antioxidants were observed to exert beneficial effects on SERCA. The antioxidant *t*-butylhydroxytoluene (BHT) or 4-hydroxy-2,2,6,6-tetramethylpiperidine-1-oxyl (TEMPOL) reversed impaired smooth muscle SERCA function in conditions of hypercholesterolemia or high glucose, and this correlated with improved relaxation to NO. Preventing SERCA from oxidation with antioxidants or protecting it against excess of reactive oxygen species (ROS) production can also slow down the irreversible oxidation of SERCA and its dysfunction (Tong *et al*., 2010).

We previously observed a protective effect of the flavonoid rutin on SERCA activity after peroxynitrite-induced injury *in vitro* (Viskupicova *et al*., 2009). Pycnogenol^®^ and EGb 761 are standardized natural plant extracts with known content of flavonoids. Pycnogenol^®^ is water extract from the bark of the French maritime pine, *Pinus pinaster*. It was found to be an efficient ROS scavenger, including hydroxyl radical (OH·) and superoxide anion radical (O_2_·^−^), as well as on NO scavenger (Virgili, 1998). EGb 761 is an extract from leaves of *Ginkgo biloba*. It is an effective free radical scavenger showing antioxidant activity and protecting against damage caused by free radicals. The methanol extract and ethyl acetate fraction obtained from yellow leaves of *Ginkgo biloba* exerted a scavenging activity of both ONOO^–^ (Hyun *et al*., 2006) and ·NO (Cheung *et al*., 1999).

In this study we assessed the potential protective effect of two natural flavonoid plant extracts EGb 761 and Pycnogenol^®^ on SERCA activity and posttranslational modifications. After treatment of SR with peroxynitrite, we determined thiol group oxidation and protein carbonyl formation.

## Materials and methods

### Sarcoplasmic reticulum isolation

SR vesicles were isolated from the white muscle in the spinal region of a New Zealand female rabbit (about 2.5 kg) according to Warren *et al*. (1974) and modified by Karlovska *et al*. (2005).

### Peroxynitrite synthesis

Peroxynitrite was synthesized by a reaction of NaNO_2_ (0.6 mol/l), H_2_O_2_ (0.7 mol/l), HCl (0.6 mol/l) and NaOH (3 mol/l) according to Radi *et al*. (1991). NaNO_2_ and H_2_O_2_ were mixed (4°C) under intensive stirring, and immediately after HCl addition NaOH was pipetted. In order to eliminate the excess of H_2_O_2,_ manganese dioxide (7 mg/ml) was added to the mixure for 1 hour at 4°C. ONOO^–^ concentration was measured spectrophotometrically.

### Ca^2+^-ATPase activity measurement

Sarcoplasmic reticulum Ca^2+^-ATPase activity was measured spectrophotometrically by NADH-coupled enzyme assay outlined by Warren *et al*. (1974) and modified by Karlovska *et al*. (2006). The final concentration of SR vesicles was 12.5 µg/cuvette. The assay mixture containing Hepes (40 mM, pH 7.2), KCl (0.1 M), MgSO_4_ (5.1 mM), ATP (2.1 mM), phosphoenolpyruvate (0.52 mM), EGTA (1 mM), NADH (0.15 mM), pyruvate kinase (7.5 IU), and lactate dehydrogenase (18 IU) was incubated at 37°C for 2 min. The reaction was started by addition of CaCl_2_ (1 mM). The reaction rate was determined by measuring the decrease of NADPH absorbance at 340 nm, at 37°C.

### Treatment with peroxynitrite and phenolic compounds

SR vesicles (0.1 mg prot./ml) from rabbit skeletal muscle were incubated for 2 min with individual antioxidants (0.5–25 µg/ml) and subsequently oxidized by peroxynitrite (100 μM) at 25°C, pH 7.2 .

### Protein carbonyl detection

Enzyme-linked immunosorbent assay(ELISA) was used for the quantitative determination of protein carbonyls according to Buss *et al*. (1997). 2,4-Dinitrophenylhydrazine (DNPH) was bound to SERCA′s carbonyl groups in dark and protein samples were absorbed in multiwell-plates (Nunc Immunosorp plates, Roskilde, Germany). Anti-dinitrophenyl-rabbit-IgG antiserum (Sigma, USA) was used as the primary antibody and monoclonal anti-rabbit-IgG-antibody (Sigma, USA) as the secondary antibody. The development of color reaction was performed using o-phenylenediamine. Absorbance was determined at 492 nm, using reference filter at 750 nm. Oxidized bovine serum albumin (BSA) was used as standard.

### SH group determination

The content of SH groups in SERCA was determined using fluorescent probe ThioGlo1 (TG1) (Sharov *et al*., 2006) and by measuring the absorbance at 513 nm with excitation at 379 nm. SR was incubated with TG1 (2 mM), SDS (20%) and PBS for 30 min, at 37°C.

## Results

The study assessed possible protective effects of natural flavonoid extracts (from leaves of *Ginkgo biloba*, EGb 761 and from the bark of *Pinus pinaster*, Pycnogenol^®^) on SR from rabbit skeletal muscle treated with peroxynitrite. SR was pretreated with individual natural extracts and afterwards incubated with peroxynitrite of IC_50_ (100 µM).

**Figure 1:**
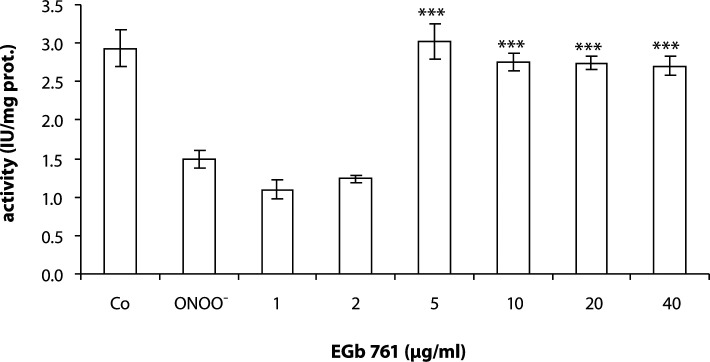
Effect of EGb 761 on SERCA activity in the presence of ONOO^–^ (100 µM). SR was exposed to various concentrations of EGB 761 for 2 min followed by oxidation by peroxynitrite at 25°C, pH 7.2. Values are mean ± SD of two independent experiments, with each sample measured in three parallels. ***p<0.001 are significant differences between oxidized sample and EGB 761 treated samples.

SR pretreated with 1 or 2 µg/ml of EGb 761 showed no protective effect on SERCA activity. However, EGb 761 in the concentration range of 5–40 µg/ml exerted a protective effect on SERCA activity injured by peroxynitrite (Figure 1).

**Figure 2:**
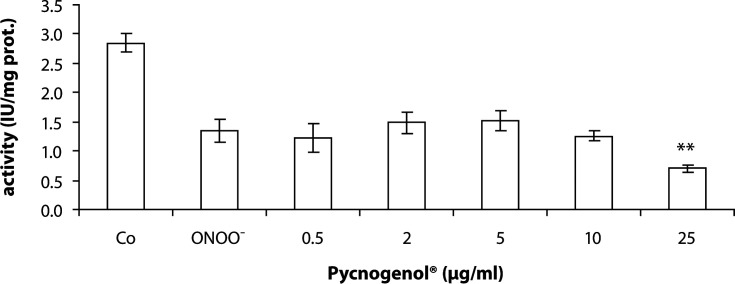
Effect of Pycnogenol^®^ on SERCA activity in the presence of ONOO^–^ (100 µM). SR was exposed to various concentrations of Pycnogenol^®^ for 2 min followed by oxidation with peroxynitrite (25°C, pH 7.2). Values are mean ± SD of three independent experiments, with each sample measured in two parallels.**p<0.01 are significant differences between oxidized sample and Pycnogenol^®^ treated samples.

Pycnogenol^®^ under the same experimental conditions had no protective effect on SERCA activity in the concentration range of 0.5–25 µg/ml. On the contrary, an additional decrease of SERCA function was observed at the concentration of 25 µg/ml (Figure 2).

In the presence of peroxynitrite concentration of 100 µM, no SH-group oxidation in SR was found (control = 13 072.67 ± 723.74 a.u., 100 µM of peroxynitrite 12 960 ± 493.65 a.u.). The peroxynitrite concentration of 500 µM caused a significant decrease (by 35%) of SH-groups (8 460.75 ± 478.17 a.u.), compared with the control (not shown). No plant extract concentration was found effective against thiol group oxidation, when SERCA was treated with peroxynitrite concentration of 500 µM.

**Figure 3:**
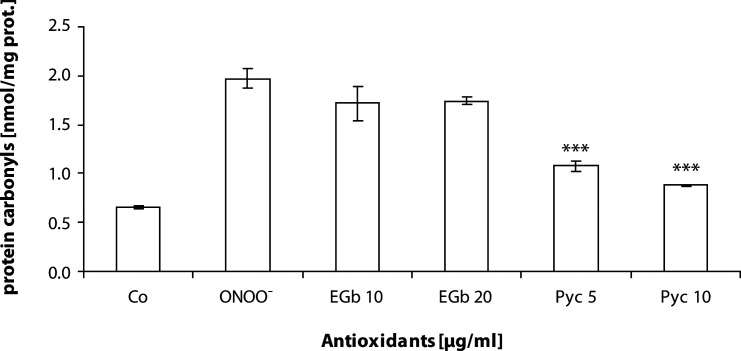
Production of protein carbonyls in pretreament of SERCA with EGb 761 and Pycnogenol^®^ in the presence of peroxynitrite. Values are mean ± SD of three independent experiments, with each sample measured in four parallels. ***p<0.0001 are significant differences between oxidized sample and flavonoid treated samples.

Protein carbonyls in SR increased approximately three times after treatment with 100 µM peroxynitrite (from 0.652 ± 0.014 a.u. to 1.974 ± 0.106 a.u.). EGb 761 in concentrations of 10 and 20 µg/ml did not prevent protein carbonyl generation in the presence of peroxynitrite. Pycnogenol^®^ in concentrations of 5 and 10 µg/ml significantly decreased protein carbonyl formation by 44% and 54%, respectively (Figure 3).

## Discussion

In SR from skeletal muscle, a high percentage of total protein content (60–90%) belongs to Ca^2+^-ATPase (SERCA) (Engelenderm *et al*., 1995; Pessah *et al*., 2002). SR vesicles from skeletal muscle are relatively simply isolable and their function can be easily measured. In addition, SERCA is the only ion transporting enzyme present in SR membrane of skeletal muscle. Nitric oxide plays a key role in the regulation of some cellular events via direct modification of SERCA protein and its injured function is associated with several diseases (Tong *et al*., 2008; Vangheluwe *et al*., 2005). Therefore we used skeletal muscle SERCA to study its injury induced by peroxynitrite *in vitro*. Since antioxidants were able to reverse impaired SERCA function in conditions of hypercholesterolemia or high levels of glucose and to improve smooth muscle response to nitric oxide (Tong, 2010; Adachi, 2002; Taguchi, 2007), our idea was to study effects of natural polyphenolic plant extracts, known as potent free radical scavengers.

It is known that cysteine residues of SERCA are extremely vulnerable and that selective and reversible oxidation of these amino acid residues is responsible for the decrease and restoration of SERCA function (Sharov *et al*., 2006; Dremina *et al*., 2007). Direct two-electron oxidation reaction of thiols with peroxynitrite results in the formation of sulphenic acid that can be stabilized or more frequently converted to disulphide (Szabo, 2007; Quijano *et al*., 1997; Carballal *et al*., 2003). Thiols can be oxidized by peroxynitrite-derived radicals and also by one-electron reactions, thus they can initiate radical-dependent chain reactions to produce higher oxidation states of sulphur, including sulphinic and sulphonic acid derivatives (Quijano *et al*., 1997; Bonini & Augusto, 2001). In this work, we found no decrease of SH groups at the concentration of 100 µM peroxynitrite, what can be explained by using DTT as reduction agent during isolation of SR. However, higher concentrations of peroxynitrite (above 250 µM) induced a decrease of SH groups, possibly caused by formation of irreversible sulphinic and sulphonic acid derivatives.

The natural polyphenolic plant extracts EGb 761 and Pycnogenol^®^ possess the ability to scavenge oxygen and nitrogen radicals (Kobuchi *et al*., 1997; Abdel-Kader *et al*., 2007; Cheung *et al*., 1999) and they are simultaneously able to bind to proteins, thus changing their function thanks to their flavonoid content (Rohdewald, 2002).

In spite of the ability of the natural plant extracts studied to scavenge both ROS and RNS, only EGb 761 exerted a protective effect on SERCA activity, while Pycnogenol^®^ failed to do so. The protective effect of EGb 761 is useful in diseases in which free radicals are involved, *e.g.* anoxia and ischemia of the brain, heart and eye, as well as atherosclerosis, rheumatism and cancer (Ellnain-Wojtaszek *et al*., 2002). The protection of EGb 761 with respect to SERCA activity did not correlate with reduction of protein carbonyls.

On the contrary, Pycnogenol^®^ was not able to prevent SERCA activity in spite of decreasing protein carbonyl generation. These results support the idea that modulation of SERCA activity by natural flavonoids may not to be associated with their antioxidant properties but rather with their ability to induce conformational changes of SERCA, resulting in changes of SERCA function (activity). The fact that EGb 761 protects and Pycnogenol^®^ inhibits SERCA activity also supports the idea that conformational alterations of SERCA are more probably involved in changes of SERCA activity. The reasons of distinguished modifications of SERCA activity by individual flavonoid extracts (EGb 761 and Pycnogenol^®^) may be revealed by detailed studies of their conformational changes in the cytosolic as well as in the transmembrane region of SERCA.
